# Early economic evaluation of the digital gait analysis system for fall prevention–Preliminary analysis of the GaitSmart system

**DOI:** 10.1002/agm2.12290

**Published:** 2024-02-07

**Authors:** Fernando Zanghelini, Georgios Xydopoulos, Richard Fordham, Geraldine Rodgers, Saval Khanal

**Affiliations:** ^1^ Health Economics Consulting, Norwich Medical School University of East Anglia Norwich UK; ^2^ NELFT NHS Foundation Trust Essex UK

**Keywords:** cost‐effectiveness analysis, early economic evaluation, feasibility analysis, GaitSmart, return on investment

## Abstract

**Objective:**

To develop an early economics evaluation (EEE) to assess the cost‐effectiveness of the GS in reducing the RoF and FoF.

**Methods:**

A cost‐effectiveness analysis (CEA) with a return on investment (RoI) estimation was performed. CEA used the most relevant parameters, such as increased gait speed and decreased FoF, to estimate the reduction in the RoF, the impact on health care resources used and financial implications for the National Health System in the United Kingdom. Outcomes were measured as incremental cost‐effectiveness ratio per quality‐adjusted life years (QALYs) gained based on the reduction of the RoF and FoF. Uncertainties around the main parameters used were evaluated by probabilistic sensitivity analysis.

**Results:**

The CEA results showed that the GS is a dominant strategy over the standard of care to improve the movements of older persons who have suffered a fall or are afraid of falling (incremental QALYs based on FoF = 0.77 and QALYs based on RoF = 1.07, cost of FoF = ‐£4479.57 and cost of RoF = ‐£2901.79). By implementing the GS, the ROI results suggest that every pound invested in the GS could result in cost savings of £1.85/patient based on the RoF reduction and £11.16/patient based on the FoF reduction. The probability of being cost saving based on the number of iterations were 79.4 percent (based on FoF) and 100 percent (based on RoF).

**Conclusion:**

The EEE supports the main hypothesis that the GS is an effective intervention to avoid falls and is potentially cost saving.

## Graphical abstract

The GaitSmart intervention, examined for cost‐effectiveness, outperforms standard care in averting falls. The GaitSmart enhances movement, contributing to increased quality‐adjusted life years and substantial cost savings. These findings affirm the effectiveness of GaitSmart in fall prevention, making it a promising strategy for improving patient outcomes and reducing health care expenses.

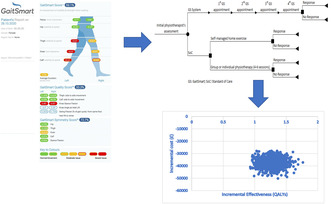

## 1 INTRODUCTION

Falls and fall‐related injuries are common and serious problems for older people. People aged 65 years and older have the highest risk of falling, with 30 percent of people older than 65 and 50 percent of people older than 80 falling at least once a year.^1^ In addition to the risk of injuries, falls may also increase the fear of falling (FoF).^2^ The FoF may result in a reduction in daily physical activity, which can lead to decreased muscle strength and an increased risk of falls (RoFs).^3^, ^4^, ^5^

Falls and their sequelae can also lead to a decreased quality of life up to 9 months after a fall, suggesting that even after this period, older adults still suffer from their falls and their consequences.^6^ It is known that the budget impact for National Health Service (NHS) in the United Kingdom due to fragility fractures is £4.4 billion, of which hip fractures alone is over £2 billion a year.^7^, ^8^ Beyond the health system costs, other costs must also be accounted for, such as individual expenses, help from family and/or friends, and productivity losses due to absence from work or unpaid activities.^9^, ^10^

Gait abnormalities are associated with functional loss and falls with important repercussions for the health of the older adults.^11^ Eighty‐two percent of the people older than 85 years old present gait abnormalities, in many cases, due to osteoarticular or neuromuscular pathologies, which are easily recognizable by clinicians.^12^ Unlike a young person, the fall of an older person can have devastating consequences for their health and quality of life. Multiplied across the population, the health and financial impacts of a fall become a major public health issue.^12^

Presently, there lacks an objective measure offering comprehensive insight into a patient's walking ability. Assessing a patient's gait speed serves as a valuable means to identify potential issues, particularly in falls in clinics addressing falls and fractures. This requires a whole system multi‐agency approach and there are currently numerous health and care organizations and professionals working with at‐risk populations. These activities need to be coordinated and overseen through effective governance.^13^

The GaitSmart (GS) system has been trialed in NHS trials involving older individuals who experienced a fall or had a moderate to severe FoF while residing in a community care unit. Utilizing an algorithm, the GS system generates detailed and objective data, offering a precise measurement of the patient's walking ability.

The economic impact of falls on older persons is an issue of growing concern to public health policymakers and clinicians as the population aged over 60 years in the United Kingdom increases.^14^ Due to the high RoFs and the fall impact on people over 65 years, falls are a good independent predictor of admission to long‐term care. Aiming to estimate the feasibility of the long‐ and short‐term impact of RoF, we developed an early economic evaluation (EEE) to estimate the cost‐effectiveness associated with using the GS system to improve movements and reduce the RoF and the FoF in older persons. Furthermore, based on the gait profile and FoF, we have calculated the rate of return on investment (RoI) of using the GS system in reducing the current care cost associated with fall incidents.

## 2 METHODS

### 2.1 Study design

An early economic model (decision tree model) and an RoI tool were developed to understand the likely cost‐effectiveness and estimate the RoI rate of using the GS system versus the standard of care (SoC) for improving movements in older persons who had fallen or had moderate to severe FoF and were in a community care unit in the United Kingdom. The economic evaluation was conducted according to the Consolidated Health Economic Evaluation Reporting Standards guidelines (CHEERS) for reporting results.^15^

### 2.2 Study population

The target population consisted of older persons who had suffered a fall or had a moderate to severe FoF and were in a community care unit. There was a cohort of 117 patients aged 79 ± 9.4 years in average with the mean body mass index of 24.9 ± 4.9. The male (45.8%) and female (54.2%) patients were approximately evenly distributed.

### 2.3 Study perspective

From the NHS perspective, the early economic model and the RoI tool were developed to determine whether the GS system is cost‐effective compared with the SoC for improving movements in those who had fallen or had moderate to severe FoF, adhering to the National Institute for Health and Care Excellence (NICE) guide to the methods of technology appraisal.^16^ Therefore, only the health care costs (direct medical costs) incurred by the provider were considered.

### 2.4 Intervention and comparator

The intervention group were the ones who were provided the GS intervention, and these were compared with the people receiving SoC treatment. The GS is a sensor‐based digital medical device (CE Marked Class 1 M Medical Device Ltd.), which has been used in clinical settings for the health rehabilitation of older persons who have suffered falls or are at risk of falling, due to different levels of frailty. The GS is worn by attaching six sensor modules to specific areas of the body using accompanying straps. The sensors, called inertial measurement units (IMUs), are synchronized using dedicated software, disconnected from the computer, and mounted into the appropriate strap pockets. The straps are applied to the lateral sides of the hip, above the iliac crest, the thigh, below the greater trochanter, and the calf's gastrocnemius muscle. The IMU system is applied over the participant's clothing as per specified location. Using an algorithm, the GS provides a detailed and objective measure of a patient's walking ability, in which the collected data are used to automatically define a personalized exercise program. All exercises were recommended either in the Otago Exercise Program (OEP) or in the NHS older people guidance, as per current appropriate practice. The OEP is considered for implementation in patients because it is one of the most beneficial programs for preventing falls.^17^ The difference between the exercises recommended by the OEP, NHS, and the GS is that the GS system only recommends exercises that focus on the specific weaknesses identified by itself. Furthermore, patients and health professionals have objective and clear data that quantify gait issues and allow them to define their own goals.

Patients assigned to the intervention group (the GS group) were from the study cohort and were monitored four times during the implementation of the intervention, 3 weeks apart. To deliver the intervention, a 10‐meter quiet (unobtrusive) straight corridor was used, and patients wore flat or low‐heeled shoes with proper support and were instructed to use the same footwear at each appointment wherever possible. All interventions were delivered by the research team. Training of the research team was carried out by Dynamic Metrics Ltd. (DML).

Patients in the SoC group were a proxy group of patients who were given advice on self‐directed rehabilitation. The SoC group consisted of those who were assessed to be at risk of falls. These individuals may be receiving treatment aimed at reducing the risk and occurrence of falls, as well as addressing the distress, pain, injuries, loss of confidence, and loss of independence associated with them. They may be receiving multifactorial interventions based on NICE Clinical Guidance (CG 161).

### 2.5 Time horizon

As the benefit of treatment is generally seen in the short term, we developed a cost‐effectiveness analysis (CEA) with a time horizon of 12 months to estimate the incremental costs and benefits of using the GS system. Given that our analytic time horizon has been 12 months, a discount rate was not applied over the costs and benefits, as recommended in the NICE reference case.^16^

### 2.6 Outcomes

Briefly, the relative effects of the GS system were focused on gait improvement in terms of changing speed and reducing FoF. Both measures were used to calculate the change in fall risk and assess their impact on fall incidents.

Comparison between both interventions (the GS system vs SoC) was performed in terms of clinical outcomes, health effects, and costs, with the clinical outcomes rated as mild or severe injuries due to a fall or multiple fall incidents in the timeframe of the model. The main outcome for CEA was the incremental cost‐effectiveness ratio (ICER) measured by the following outcomes: change in gait speed and reduction in FoF.

### 2.7 Model structure

**FIGURE 1:**
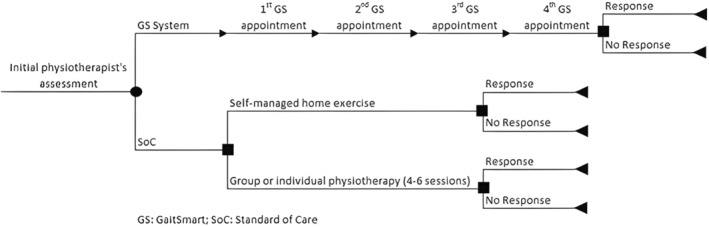
Decision tree model of the GaitSmart system.

An analytic decision tree model has been developed in Microsoft Excel 2013 to compare the costs and benefits (effectiveness) of the current SoC pathway vs the new pathway with the introduction of the GS intervention. The Figure 1 shows how a cohort of patients who had suffered a fall or had a moderate to severe FoF might move through the hypothetical decision tree over a 12‐month time horizon.

Subjects assigned to the GS group were monitored four times during intervention implementation, 3 weeks apart, and compared to the SoC group, treated following NICE Guidelines.^18^ Patients assigned to receive SoC were not monitored with the GS system and could be allocated to receive either self‐managed home exercise or group or individual physiotherapy (4–6 sessions). At the end of the path, each branch of the decision tree provides the outcomes of the model (response or no response).

Moreover, the model considers the rates of falling incidents within the model's timeframe, as reported in the literature. It links the different levels of patient speed before and after a cycle of four GS sessions to changes in the risk rate, based on findings from previous studies. This results in a personalized calculation for patients, estimating the total reduction in the risk of falling associated with the cohorts under examination. The model also performs a second calculation of the fall incidents based on the improvement of FoF. Data related to FoF were collected using the Falls Efficacy Scale International (FES‐I) tool before and after four GS test sessions. The fall incidents were then split into injurious falls and falls that lead to no harm, based on parameters extracted from the literature, because there were no fall incidents during the subjects’ short‐term follow‐up. Evidence on FoF and its correlation with falling incidents was gathered from the literature, as a substantial proportion of people who fall lose their independence and FoF can increase the risk of further falls.

## 3 MODEL INPUT PARAMETERS

### 3.1 Clinical effectiveness

The European Technologies for Business Holdings provided data on 86 frail older adults (average age of 80 years) from the North East London Foundation Trust (NELFT) who had previously fallen and were under the care of the Community Hospital. These data included age, walking aids used, fall incidents experienced, FoF data, FES‐I data, and cost of the intervention. Gait data were collected on four different occasions/tests during the implementation of the intervention and the walking speed (in meter/second), overall gait score based on the GS outputs, and FES‐I of the participants were determined at the start and end of the intervention. Each participant did provide written informed consent. This was a quality improvement program run by the NHS and did not require ethical approval.

The relative RoF for the subjects assessed at a specific speed level was extracted from the literature as well as the correlation between improving gait speed by 0.1 meters per second (m/s) and the reduction in relative risk.^13^, ^14^, ^15^, ^16^ This allowed for the calculation of a personal risk profile for the initial state of each participant and to calculate their risk of falling reduction based on their speed improvement.

Data related to a person's FoF were collected from the literature and separated into three levels, “’not afraid’,” “’moderately afraid,”’ and “’very afraid’.” Published data also provided the correlation between FoF and the number of falls and injuries that occurred in the following year.^2^, ^19^, ^20^

### 3.2 Costs

The EEE was undertaken from the NHS perspective, using the national annually published resource, the Personal Social Services Research Unit, and the Unit Costs of Health and Social Care 2018.^21^ Briefly, the analysis only accounts for direct medical costs, non‐health‐related costs due to lost productivity and informal care are not included. The model progressively calculates the cost of falls, estimating the reduction of RoF and the reduction of FOF in the selected population, based on trial data and literature parameters. Costs of health resources, such as GP appointments, accidents and emergencies admission, inpatient treatment, ambulance call, and length of stay were expressed in British Pounds 2018 (£), and an overview of resource use and values used in the economic assessment are shown in Table 1. They are also presented in the File S1 in more detail.

**TABLE 1:** Model input parameters.

Input parameters group	Input parameters	Deterministic	Probabilistic	Distribution	Resources
General parameters	Transition probabilities	Values (RR)			
	Percentage of community dwelling adults over 65 y that will experience a fall each year	0.333	0.35	Beta	Berry et al., 2008; Tinetti et al., 1995
	Percentage of institutionalized adults over 80 y that will experience a fall each year	0.50	0.47	Beta	
	Percentage of falls that result in an injury that requires medical attention	0.20	0.22	Beta	
	Percentage of persons over 70 y old that will go to A&E after a fall	0.080	0.11	Beta	
	Percentage that result in fractures	0.150	0.14	Beta	
	Percentage that result in head trauma/serious injury	0.340	0.34	Beta	
	Percentage that result in injury requiring medical attention	0.20	0.22	Beta	
	Percentage that result in minor injuries	0.333	0.31	Beta	
	Falls that will result in A&E attendance	0.340	0.29	Beta	
	Falls that will result in GP visit	0.510	0.51	Beta	
	Falls that will result in ambulance call out	0.150	0.17	Beta	
Fear of falling level and probabilities of experience a fall or recurrent falls per fear of falling level	Low fear of falling	0.6685	0.7833	Calculated	FES‐I questionnaire
	Moderate fear of falling	0.2852	0.1862	Beta	
	High fear of falling	0.0463	0.0356	Beta	
	At least 1 fall (low fear of falling)	0.260	0.201	Beta	Arfken et al., 1994
	At least 1 fall (moderate fear of falling)	0.360	0.357	Beta	
	At least 1 fall (high fear of falling)	0.480	0.443	Beta	
	Recurrent fall (low fear of falling)	0.080	0.717	Beta	
	Recurrent fall (moderate fear of falling)	0.130	0.100	Beta	
	Recurrent fall (high fear of falling)	0.220	0.183	Beta	
Costs	GP cost per average appointment (10 min)	£ 36.00	£ 38.21	Gamma	Public Health England, 2018
	A&E attendance – no admission	£ 100.53	£ 101.29	Gamma	Public Health England, 2018
	Non‐elective inpatients	£ 1609.00	£ 1557.02	Gamma	Curtis, L. & Burns, A., 2016
	Ambulance call‐out	£ 236.00	£ 250.55	Gamma	Public Health England, 2018
	Excess length of stay cost per day	£ 306.00	£ 298.26	Gamma	Curtis, L. & Burns, A. 2016
	GS total cost per patient	£ 40	–	–	Study assumption

Abbreviations: A&E, accidents and emergencies; FES‐I, Falls Efficacy Scale International; GP, general practitioner; GS, GaitSmart; RR, risk ratio.

### 3.3 Utility values

The utility values assigned to responders and nonresponders for the GS and SoC interventions were imputed from the literature based on the expected fall incidents per RoF or FoF level, at the start and end of the intervention implementation.^21^, ^22^, ^23^ The utility value derived from the literature and the calculation are provided in more detail in File S2.

### 3.4 Modeling assumptions

In this study, we assumed a time horizon of 1 year. Due to the lack of a randomized clinical trial setting with a comparator and the study population monitored for a short follow‐up period, we relied on published data about the risk of falling based on specific fear of falling levels. We then utilized a proxy comparator approach based on the original risk of falling. The utility data for these comparators were obtained from the published literature.^21^, ^22^, ^23^ We carefully selected the closest utility values that matched our cohort.

### 3.5 Sensitivity analysis

The impact of uncertainties around the model's key parameters on the ICER value was assessed through 1000 probabilistic sensitivity analysis (PSA) using Monte Carlo simulation. These analyses consisted of varying each key parameter based on its distribution.^24^, ^25^, ^26^ The scatter plot of the ICER of the GS system vs SoC and cost‐effectiveness acceptability curves (CEACs) was created from these analyses. In addition, a one‐way deterministic threshold analysis was performed on the cost of the GS system to find the price at which the intervention would no longer be cost saving.^26^

### 3.6 Return on investment

The RoI aims to assess the investment performance made. The RoI shows until which point the amount invested in this given health technology returns as profit or loss. Therefore, it allows the effectiveness assessment of resources invested. The RoI is calculated as the ratio between profit obtained after investing and the total investment costs. As the result is a percentage, the ratio obtained should be multiplied by 100, as the following formula:

$$\mathrm{ROI}=\frac{\text{Treatment Costs Saved}}{\text{Program Investment}}$$

Where,

Treatment costs saved = [Cost of Falls (CP) + Physio Costs (CP)]‐[Cost of Falls (IP) + Physio Costs (IP)] *(Note: CP = Conventional pathway, IP = Intervention Pathway)*.

Program Investment = Intervention cost.

## 4 RESULTS

### 4.1 GaitSmart system effectiveness

The personalized risk profile created for each participant based on the improvement in their gait speed shows a reduction in the average RoF by 1.77 percent (average initial risk 0.5, risk after intervention 0.482). Sixty‐nine of the 86 patients (patients in the NELFT cohort) have improved their walking speed after four GS sessions. The greatest improvement in gait speed was more than three times the initial gait speed, representing a 9.77 percent reduction in the RoF. Whereas the smallest improvement was two percent in the initial speed, representing a 0.13 percent reduction in the RoF. For the remaining 17 patients without documented improvement, the greatest increase in the RoF was 2.3 percent.

Regarding the change in FoF, 45 percent of participants showed a decrease in the level of FoF after receiving the intervention (the GS group), and 11.49 percent of participants changed from a high‐level FoF to a low level.

### 4.2 Base case

At the end of 12 months, the CEA results showed that patients assigned to the GS intervention had higher improvement in their quality of life (incremental quality‐adjusted life years [QALYs] based on FoF = 0.77 and incremental QALYs based on RoF = 1.07) compared to those assigned to receive SoC intervention. Furthermore, the GS system was less costly for the outcomes assessed (cost of FoF = ‐£4479.57 and cost of RoF = ‐£2901.79). Therefore, the GS system is a dominant strategy over the SoC to improve the movements of older persons who have suffered a fall or are afraid of falling, increasing the quality of life and generating cost savings (Table 2).

**TABLE 2:** Cost‐effectiveness analysis results.

**SoC**									
	Cost		Effectiveness		Incremental				ICER
	Expected costs FoF (£)	Expected costs RoF (£)	QALYs based on FoF	QALYs based on RoF	Cost FoF (£)	Cost RoF (£)	QALYs FoF	QALYs RoF	ICER (£)
Deterministic results	15,363.65	119,152.27	685.08	667.45	–	–	–	–	–
Probabilistic results	15,482.58	153,957.27	685.06	663.54	–	–	–	–	–
L95CI	8301.37	123,767.33	683.46	657.57	–	–	–	–	–
U95CI	25,849.17	186,201.04	686.18	668.70	–	–	–	–	–
**GS intervention**									
Deterministic results	10,884.08	116,250.48	685.85	668.52	−4479.57	−2901.79	0.77	1.07	Dominant
Probabilistic results	11,081.06	116,956.82	685.82	664.82	−4401.52	−37,000.45	0.76	1.28	Dominant
L95CI	3547.20	93,467.44	683.86	659.07	−4754.17	−30,299.89	0.40	1.50	
U95CI	23,352.87	142,201.28	686.97	669.79	−2496.3	−43,999.76	0.79	1.09	

Abbreviations: FoF, fear of falling; GS, GaitSmart; ICER, Incremental Cost‐Effectiveness Ratio; L95CI, lower 95% confidence interval; QALYs, Quality Adjusted Life‐Years; RoF, risk of fall; SoC, standard of care; U95CI, upper 95% confidence interval.

### 4.3 Sensitivity analysis

**FIGURE 2:**
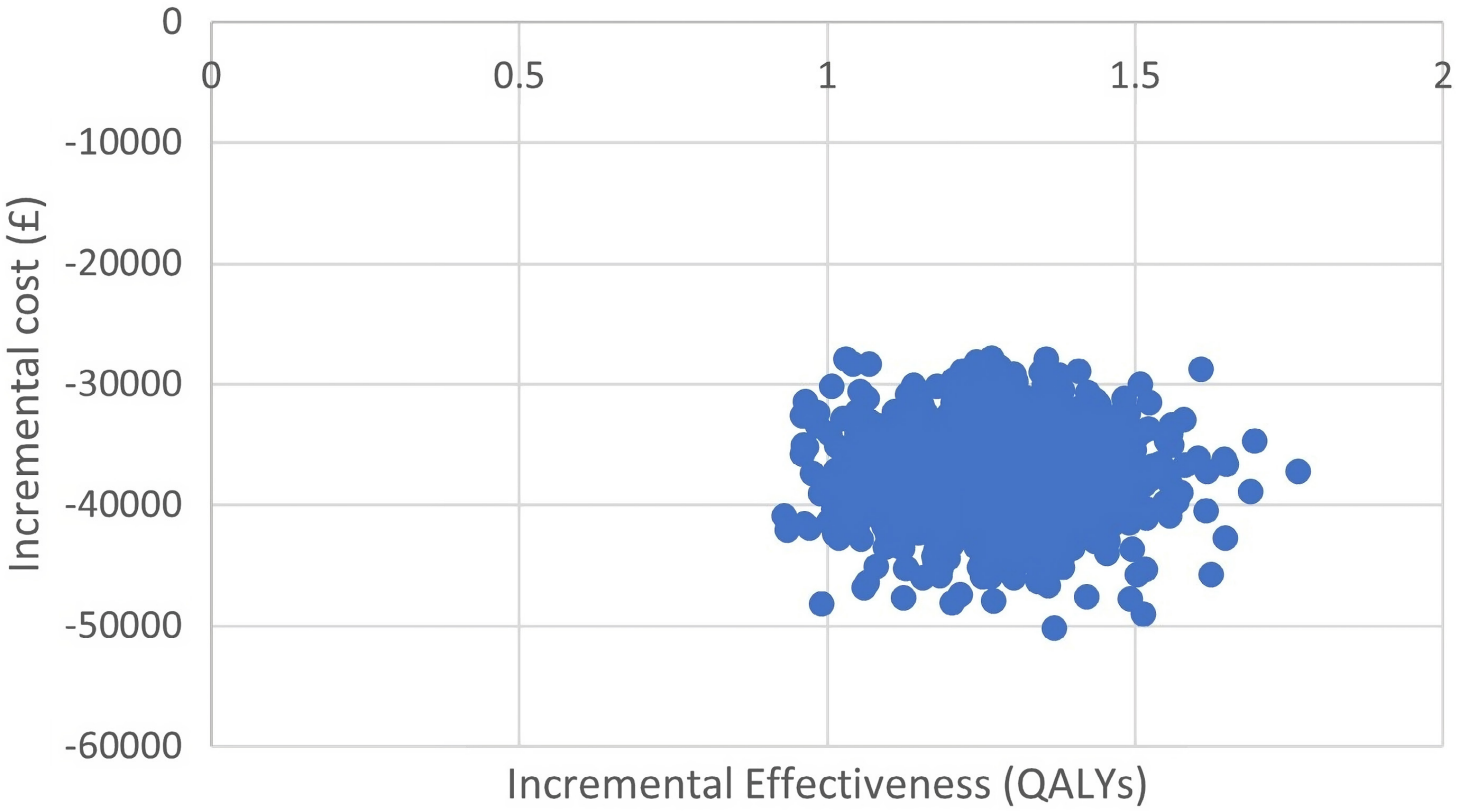
Scatter plot of the incremental cost‐effectiveness ratio of the GS system versus standard of care. QALYs, Quality Adjusted Life‐Years.

Considering the inherent uncertainties associated with the input values integrated into the model, a PSA was conducted. Figure 2 delineates the scatter plot of incremental cost‐effectiveness, thereby elucidating the robustness of the results. All simulated scenarios are distinctly situated in the southeast quadrant, conclusively affirming the superior efficacy and reduced cost associated with the GS system relative to the SoC. Further details on the PSA results are described in Table 2 and confirm that the GS system is a dominant strategy to improve the movements of older persons who have suffered a fall or are afraid of falling. The probability of intervention being cost‐effective based on the PSA results were 79.4 percent (FoF) and 100 percent (RoF).

The CEAC (File S3) for the scenario studied indicate the probability of the intervention being cost‐effective when compared to the alternatives, according to the different thresholds. In all cases, the curves support the results suggested by the scatter plot. When comparing the GS system with SoC all scenarios show that the GS system is a cost‐effective option regardless of the willingness‐to‐pay threshold.

### 4.4 Return on investment

An RoI was subsequently calculated (Table 3). The net RoI suggests that every pound invested in the GS sytem compared to the SoC results in cost savings of £1.85 per patient based on the RoF reduction calculations and £11.16 per patient based on the FoF reduction assessment. An RoI lower than one indicates that the program costs more to deliver than was saved in terms of treatment costs. An RoI greater than one indicates the program has saved enough in terms of treatment costs to more than cover its costs. Therefore, the GS system presents a positive RoI alongside conventional physiotherapy and improves physiotherapist productivity. If the GS system were used as an alternative to conventional physiotherapy, the RoI would be even higher.

**TABLE 3:** The GS system return on investment per percentage of reduction in physiotherapy costs.

Percentage reduction in physiotherapy costs	GS RoI (RoF)	GS net RoI (RoF)	GS RoI (FoF)	GS net RoI (FoF)
0%	£1.85	£0.85	£11.16	£10.16
1%	£2.03	£1.03	£11.29	£10.29
3%	£2.39	£1.39	£11.55	£10.55
5%	£2.76	£1.76	£11.81	£10.81

Abbreviations: FoF, fear of falling; GS, GaitSmart; RoF, risk of falling; RoI, return on investment.

## 5 DISCUSSION

In all countries, policymakers face challenges on how to allocate scarce resources for health. CEA provides a means of comparing health costs and gains from interventions as a basis for informing investment decisions and informing evidence‐informed policies.^27^ A decision tree analysis with a time horizon of 12 months was developed to estimate the potential results of implementing the GS system alternative to current SoC. Based on our predefined parameters, this study showed that the GS system has the potential to be the most cost‐effective intervention for reducing falls and fear of falling in older adults. It has the potential to be a cost‐effective measure within the NHS, either as a standalone intervention or in combination with other approaches, like medications, nutrition therapy, psychological therapy, environmental/assistive technology modifications, and knowledge education interventions. The GS system improved both RoF and FoF outcomes, reducing them by 1.77 percent (95 percent confidence interval [CI] = 1.05 percent to 2.67 percent) and 45 percent (95 percent CI = 41.93 percent to 48.09 percent), respectively, compared to the SoC. Previous studies have evaluated the walking speed of individuals of different ages, and their results show that when this speed deviates from the normal pattern, this represents a potential problem related to walking. All studies nominally agreed that a change of 0.05 meters/second (m/s) has a significant impact on the risk of falls and well‐being in older adults, also stating that for those with gait issues, improved speed should be greater than 0.1 m/s.^28^, ^29^, ^30^, ^31^

Hospital costs are not the only components of care that arose because a substantial proportion of people who fall lose their independence and an FoF can lead to a greater risk of future falls which require further resources from many different stakeholders, including families and caregivers, the NHS, and local authorities. Several interventions have been shown to be effective in preventing falls, in particular, interventions that contain challenging balance and functional elements result in the most beneficial outcomes.^32^, ^33^

One of the strengths of this study is that it used the most relevant parameters, such as increased gait speed and decreased FoF, to estimate the cost‐effectiveness and RoI rate of using the GS system on the RoF reduction. This is the first UK‐specific study and the first internationally to develop a novel health economics model to assess the cost‐effectiveness of the GS system in reducing the RoF and calculate the RoI using the NHS perspective. The analysis aimed to translate the clinical improvements observed in the data into tangible economic benefits for health care payers. For that, an exploratory economic model sophisticated enough to carry out an economic assessment in an area where both physical and psychological parameters influence the ability to walk confidently was developed. Hence, modeling the costs and effectiveness of reducing the risk of falling based on gait and fear of falling measurements is a feasible technique. Although it is an area covered with some ambiguity around the fear of falling metrics our experience shows that it was possible combining robust quantitative and qualitative metrics.

Regarding the GS system study preliminary data, the outcomes of the analysis show that the GS system intervention may be cost‐effective compared to the current SoC. At a time when falls are estimated to cost the NHS more than £4.4 billion per year, the GS intervention could potentially have a significant impact on improving the cost‐efficiency of health provision. The GS system has demonstrated promising early acceptability among both patients and service providers. Patients find the wearable sensor system convenient to use, as it involves attaching the sensor modules to specific areas of the body using straps. The noninvasive nature of the device, applied over clothing, further enhances its acceptability. Service providers may appreciate the system's ability to deliver accurate gait analysis and valuable insights for treatment planning. Training can be provided for service providers and ongoing support for any technical or logistical challenges that may arise. Continuous monitoring and feedback from patients and service providers can contribute to refining and optimizing the GS system during the scaling process.

A limitation of simulation models in complex areas, such as health and health care, are that they rely on many assumptions about important, but unknown parameters. Although the main data sources for the analysis are robust, there are relatively limited follow‐up data on the effectiveness of each intervention. Consequently, the timeframe of the analysis has been limited to 1 year with the assumption made that all benefits from the intervention will cease by the end of this 1 year. Clearly, this is not always the case. Furthermore, the data should be considered in terms of generalizability. The distinction between the characteristics of the study participants also limits the generalizability of the results of this analysis. Therefore, they only remain valid if the intervention is targeted at populations that are like the study cohorts.

The results are valid for the intervention targeted for populations similar to the study cohorts. However, it is expected that there will be a degree of variation in local practice, and this will impact the results of the analysis. In particular, the location is expected to impact the cost of implementing the intervention (eg, exercise classes may be more expensive to implement in rural locations due to a need for staff to travel greater distances) and the costs related to falls may differ across Integrated Care Boards.

Furthermore, long‐term fall incidents' costs and consequences were not included as this would require further assumptions to be made in the lack of long‐term participant monitoring by the study. If these costs were included, the cost‐effectiveness of screening with the intervention would potentially improve. As the model does not consider social care costs when a broader societal perspective is adopted and the impact of any intervention on a participant's quality of life is formally quantified, then returns with each intervention increase. However, if the GS system were introduced into the lower‐risk population, the expected return on investment would be different. In addition, we could not perform the subgroup analyses based on the gender as we acknowledge there may be different falls related risk and effectiveness associated with different genders, thus different returns.

Future studies have been planned to address the gaps and limitations of the current preliminary analysis, examining in more detail the time of the intervention and the comparator, thus improving the quality of available data.

## 6 CONCLUSION

The economic analysis presented implies that, in light of the current assessed patterns, opting for the GS system could be a dominant (more effective and less costly) strategy compared to the SoC. Thus, based on the results found, our model suggests that implementing the GS system for older individuals who have experienced a fall or have a fear of falling has the potential for cost savings.

Although the GS system has shown very positive cost‐efficiency outcomes, this should be taken with caution due to the limitations of the current study. It should be noted that it is expected that only a small proportion of these financial returns will be due to cash‐releasing savings. Most of the financial returns presented by the model are due to opportunity cost savings, such as the freeing up of hospital beds due to fewer inpatient admissions. Although these benefits will not necessarily produce cash that can be spent elsewhere, this will help relieve certain pressures on the NHS. Future prospective studies are needed to elucidate the uncertainties presented and improve the strength of the evidence.

## AUTHOR CONTRIBUTIONS

F.Z., G.X., R.F., and G.R. conceptualized the model. F.Z. developed initial model and then was validated by G.X. and S.K. F.Z. and G.X. identified the model inputs, and S.K. updated them during the revision. F.Z. wrote the first version of the manuscript and all authors contributed to finalize the manuscript. All authors revised and agreed to submit the current version of the manuscript.

## FUNDING INFORMATION

No funding was received for this study.

## CONFLICT OF INTEREST STATEMENT

The authors declare no conflict of interest.

## ETHICS STATEMENT

This was an evaluation work and was solely performed using available data from the secondary sources or routinely collected data, so no ethical clearance was needed.

## Supporting information


File S1.

File S2.

File S3.
