# Supplementary material for: Early economic evaluation of the digital gait analysis system for fall prevention–Preliminary analysis of the GaitSmart system
Source: Aging Med (Milton). 2024 Feb 7;7(1):74–83. doi: 10.1002/agm2.12290 (PMC10985772; doi:10.1002/agm2.12290)
Supplement: Supplementary file 1 — File S1. [file AGM2-7-74-s003.docx]

**Supplementary File 1**

**Table: Cost, and Fear and Risk of Fall inputs**

| **Models inputs** | **Full references (please provide details, e.g., HRG code, band etc)** | **Inflated (Y/N)? Please provide the cost year if inflated.** |
| --- | --- | --- |
| *Model 1 – fall prevention* | | |
| 1. Costs – ambulance call out | PSSRU 2018 pg 89 See and treat and convey (including carbon 59 kgCO2e | N |
| 1. Costs – A&E attendance, no admission | PHE, 2018. A Return on Investment Tool  for the Assessment of Falls  Prevention Programmes for Older  People Living in the Community | N |
| 1. Costs – A&E attendance, admission | PHE, 2018. A Return on Investment Tool  for the Assessment of Falls  Prevention Programmes for Older  People Living in the Community | N |
| 1. Costs – GP visit | PHE, 2018. A Return on Investment Tool  for the Assessment of Falls  Prevention Programmes for Older  People Living in the Community | Y to 2018 using Hospital & community health services (HCHS)Pay & prices index |
| 1. Costs – NHS reference cost | PSSRU 2018 pg 89 Weighted average of all outpatient attendances (inc. carbon 32 kgCO2e) | N |
| 1. Costs – Non-Elective Inpatients | It should be £1841 based on pssru 2018 non Non-elective inpatient stays (average of long and short stay) | N |
| 1. Costs – Non-Elective Inpatient Excess Bed Days | NHS Reference Costs 2016/2017 – Index - Non-elective inpatient excess bed day cost. Weighted average of all admission types. | N |
| 1. Various fall inputs - Berry 2008 | Berry, S.D., Miller, R.R., 2008. Falls: Epidemiology, pathophysiology, and relationship to fracture. Current Osteoporosis Reports. doi:10.1007/s11914-008-0026-4 |  |
| 1. Various fall inputs - Tinetti 1995 | Tinetti, M.E., Doucette, J., ... Marottoli, R., 1995. Risk Factors for Serious Injury During Falls by Older Persons in the Community. Journal of the American Geriatrics Society 43, 1214–1221. doi:10.1111/j.1532-5415.1995.tb07396.x |  |
