# Supplementary material for: Early economic evaluation of the digital gait analysis system for fall prevention–Preliminary analysis of the GaitSmart system
Source: Aging Med (Milton). 2024 Feb 7;7(1):74–83. doi: 10.1002/agm2.12290 (PMC10985772; doi:10.1002/agm2.12290)
Supplement: Supplementary file 2 — File S2. [file AGM2-7-74-s002.docx]

**Supplementary File 2**

| **QALYs General Parameters** | | | | | | | | |
| --- | --- | --- | --- | --- | --- | --- | --- | --- |
|  |  |  |  |  |  |  |  |  |
| **Utility with a hip fracture** | | **0.582** |  |  |  |  |  |  |
| **Utility with a non-hip fracture** | | **0.699** |  |  |  |  |  |  |
|  |  | |  |  |  |  |  |  |
| **Age** | **Quality of Life** | | **Penalty of Hip Fracture** | | **Penalty of a non-hip fractured injury** | | |  |
| 60-64 | 0.829 | | -0.346522 | | -0.249529 | | |  |
| 65-69 | 0.806 | | -0.336908 | | -0.242606 | | |  |
| 70-74 | 0.747 | | -0.312246 | | -0.224847 | | |  |
| 75-79 | 0.731 | | -0.305558 | | -0.220031 | | |  |
| 80-85 | 0.699 | | -0.287375 | | -0.2069375 | | |  |
| 85+ | 0.676 | |  |  |  |  |  |  |
| Average | 0.748 | | -0.312664 | | -0.225148 | | |  |

| **QALYs Based on Risk of Falling Calculations** | | | | | | | | |
| --- | --- | --- | --- | --- | --- | --- | --- | --- |
|  |  |  |  |  |  |  |  |  |
| **Conventional Pathway** | | | | | | | | |
| **Age** | **Falls** | **Recurrent Falls** | **IN F/RF** | **SIN F/RF** | **MIN F/RF** | **Start QALYs** | **End QALYs** | **Qalys diff.** |
| 65-69 | 0.00 | 0.00 | 0 / 0 | 0 / 0 | 0 / 0 | 0.00 | 0.00 | 0.00 |
| 70-74 | 0.00 | 0.00 | 0 / 0 | 0 / 0 | 0 / 0 | 0.00 | 0.00 | 0.00 |
| 75-79 | 0.00 | 0.00 | 0 / 0 | 0 / 0 | 0 / 0 | 0.00 | 0.00 | 0.00 |
| 80+ | 333.30 | 47.77 | 66.66 / 9.55 | 10 / 1.43 | 22.66 / 3.25 | 687.50 | 667.45 | 20.05 |
| All Ages | 0.00 | 0.00 | 0 / 0 | 0 / 0 | 0 / 0 | 0.00 | 0.00 | 0.00 |
| Adm. Pop. | 0.00 | 0.00 | 0 / 0 | 0 / 0 | 0 / 0 | 0.00 | 0.00 | 0.00 |
|  |  |  |  |  |  |  |  |  |
| **Intervention Pathway** | | | | | | | | |
| **Age** | **Falls** | **Recurrent Falls** | **IN F/RF** | **SIN F/RF** | **MIN F/RF** | **Start QALYs** | **End QALYS** | **Qalys diff.** |
| 65-69 | 0.00 | 0.00 | 0 / 0 | 0 / 0 | 0 / 0 | 0.00 | 0.00 | 0.00 |
| 70-74 | 0.00 | 0.00 | 0 / 0 | 0 / 0 | 0 / 0 | 0.00 | 0.00 | 0.00 |
| 75-79 | 0.00 | 0.00 | 0 / 0 | 0 / 0 | 0 / 0 | 0.00 | 0.00 | 0.00 |
| 80+ | 316.31 | 45.34 | 63.26 / 9.07 | 9.49 / 1.36 | 21.51 / 3.08 | 687.50 | 668.47 | 19.03 |
| All Ages | 0.00 | 0.00 | 0 / 0 | 0 / 0 | 0 / 0 | 0.00 | 0.00 | 0.00 |
| Adm. Pop. | 0.00 | 0.00 | 0 / 0 | 0 / 0 | 0 / 0 | 0.00 | 0.00 | 0.00 |
|  |  |  |  |  |  |  |  |  |
| Total QALYs IP | 668.47 |  |  |  |  |  |  |  |
| Total QALYs CP | 667.45 |  |  |  |  |  |  |  |
| **IP Vs CP** | 1.02 |  |  |  |  |  |  |  |

| **QALYs Based on Fear of Falling Calculations** | | | | | | | | |
| --- | --- | --- | --- | --- | --- | --- | --- | --- |
|  |  |  |  |  |  |  |  |  |
| **Conventional Pathway** | | | | | | | | |
| **Age** | **Falls** | **Recurrent Falls** | **IN F/RF** | **SIN F/RF** | **MIN F/RF** | **Start QALYs** | **End QALYS** | **Qalys diff.** |
| 65-69 | 0.00 | 0.00 | 0 / 0 | 0 / 0 | 0 / 0 | 0.00 | 0.00 | 0.00 |
| 70-74 | 0.00 | 0.00 | 0 / 0 | 0 / 0 | 0 / 0 | 0.00 | 0.00 | 0.00 |
| 75-79 | 0.00 | 0.00 | 0 / 0 | 0 / 0 | 0 / 0 | 0.00 | 0.00 | 0.00 |
| 80+ | 38.40 | 16.90 | 7.68 / 3.38 | 1.15 / 0.51 | 2.61 / 1.15 | 687.50 | 685.08 | 2.42 |
| All Ages | 0.00 | 0.00 | 0 / 0 | 0 / 0 | 0 / 0 | 0.00 | 0.00 | 0.00 |
| Adm. Pop. | 0.00 | 0.00 | 0 / 0 | 0 / 0 | 0 / 0 | 0.00 | 0.00 | 0.00 |
|  |  |  |  |  |  |  |  |  |
| **Intervention Pathway** | | | | | | | | |
| **Age** | **Falls** | **Recurrent Falls** | **IN F/RF** | **SIN F/RF** | **MIN F/RF** | **Start QALYs** | **End QALYS** | **Qalys diff.** |
| 65-69 | 0.00 | 0.00 | 0 / 0 | 0 / 0 | 0 / 0 | 0.00 | 0.00 | 0.00 |
| 70-74 | 0.00 | 0.00 | 0 / 0 | 0 / 0 | 0 / 0 | 0.00 | 0.00 | 0.00 |
| 75-79 | 0.00 | 0.00 | 0 / 0 | 0 / 0 | 0 / 0 | 0.00 | 0.00 | 0.00 |
| 80+ | 26.59 | 11.70 | 5.32 / 2.34 | 0.8 / 0.35 | 1.81 / 0.8 | 687.50 | 685.82 | 1.68 |
| All Ages | 0.00 | 0.00 | 0 / 0 | 0 / 0 | 0 / 0 | 0.00 | 0.00 | 0.00 |
| Adm. Pop. | 0.00 | 0.00 | 0 / 0 | 0 / 0 | 0 / 0 | 0.00 | 0.00 | 0.00 |
|  |  |  |  |  |  |  |  |  |
| Total QALYs IP | 685.82 |  |  |  |  |  |  |  |
| Total QALYs CP | 685.08 |  |  |  |  |  |  |  |
| **IP Vs CP** | 0.74 |  |  |  |  |  |  |  |
