# Supplementary figures and images for: Early economic evaluation of the digital gait analysis system for fall prevention–Preliminary analysis of the GaitSmart system
Source: Aging Med (Milton). 2024 Feb 7;7(1):74–83. doi: 10.1002/agm2.12290 (PMC10985772; doi:10.1002/agm2.12290)

**Supplementary File 3.**

Figure: Cost-effectiveness acceptability curves of GS system versus SoC


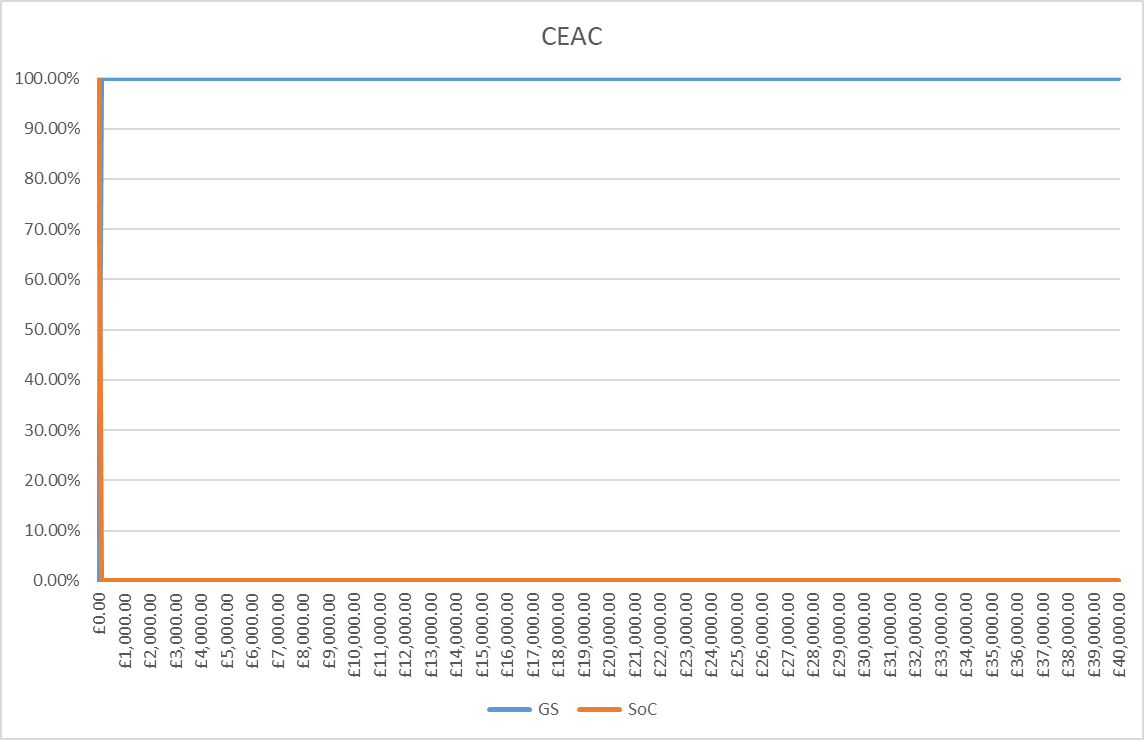

Supplement: Supplementary file 3 — File S3. [file AGM2-7-74-s001.docx]
